# GASTRIC PERFORATION ASSOCIATED WITH ACUTE PANCREATITIS: CASE REPORT

**DOI:** 10.1590/0102-6720201700020019

**Published:** 2017

**Authors:** Hector LOSADA, Maité LÓPEZ, Pablo AVARIA, Andrés TRONCOSO

**Affiliations:** 1 Universidad de La Frontera, Hepato-Pancreatic and Biliary Surgery, Temuco, IX Region, Chile;; 2 Clinica Alemana, Radiology Department, Temuco, IX Region, Chile;; 3 Clinica Alemana, Hepato-Pancreatic and Biliary Surgery, Temuco, IX Región, Chile.

**Keywords:** Pancreatitis, Acute necrotizing, Stomach rupture, Surgery.

## INTRODUCTION

Acute pancreatitis(AP) have a high morbidity and mortality^[1]^. Gastric perforation is a rare complication of AP.

The aims of the present paper were: 1) report a clinical case with a rare presentation of AP (hematemesis) with a slow evolution towards gastric perforation that was remarkable for the absence of celiac axis thrombosis as evidenced by imaging; and 2) review AP in terms of clinical presentation, imaging, risk factors, complications and treatment.

## CASE REPORT

**FIGURE 1:**
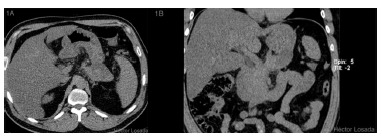
A) Axial unenhanced abdominal CT with collection contacting the posterior gastric Wall; B) coronal unenhanced abdominal CT with collection contacting the posterior gastric wall.

43-year-old male with intense epigastric abdominal pain with an episode of hematemesis looked for medical assistence. He was with heart rate of 91 bpm, blood pressure of 150/69 mmHg, temperature of 37.3° C, pale skin and mucous membranes, soft abdomen sensitive at the epigastrium. The laboratory examination results were: leukocytes: 6,080 cells/mm^3^, hematocrit 43.8%, hemoglobin 15.5 gr/dl, C-reactive protein 2.3 mg/dl, creatinine 2.18 mg/dl, amylase 168 U/l, lipase 53 U/l, and normal liver tests, plasma electrolytes and coagulation tests. An unenhanced abdominal and pelvic CT showed mild pancreatic tail enlargement, increased attenuation of the peripancreatic fat, and a pancreatic-peripancreatic collection contacting the posterior gastric wall (Figure 1A and B). 24 h after admittance follow-up analysis were: leukocytes 17,830 cells/mm^3^, hematocrit 41.7%, hemoglobin 14.6 gr/dl and C-reactive protein 36.96 mg/dl. Due to the hematemesis, was done an upper GI endoscopy that revealed diffuse gastritis with necrotic foci.

The patient’s abdominal pain persisted with peritoneal irritation signs, and follow-up laboratory examinations revealed: leukocytes 9,999 cells/mm^3^, hematocrit 44.4%, hemoglobin 15.3 gr/dl, C-reactive protein 457.1 mg/dl, amylase 616 U/l, lipase 698 U/l, total bilirubin 2.44 mg/dl, GOT/GPT 64/48 U/l and normal electrolytes. Patient had progressive hemodynamic instability, oliguria, with increased creatinine, requiring high doses noradrenaline.

A diagnosis of AP was made and he was re-evaluated, with diffuse abdominal pain with peritoneal irritation and 34 mmHg intra-abdominal pressure. Severity score of acute pancreatitis was APACHE II 10, PCR 457 ; Marshall of 4.

**FIGURE 2:**
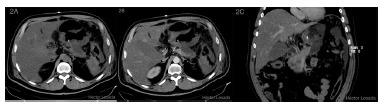
A) Axial unenhanced abdominal CT with no enhancement of body and tail of the pancreas; B) axial enhanced abdominal CT with no enhancement of body and tail of the pancreas; C) coronal enhanced abdominal CT with thickened unenhanced gastric wall and collection.

**FIGURE 3:**
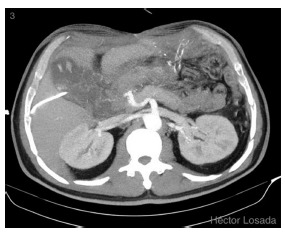
Angio-CT, axial MIP, with no vascular involvement

**FIGURE 4:**
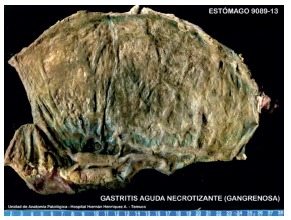
Resection specimen: gastric gangrene due to necrotizing gastritis

AP, gastric necrosis and abdominal compartment syndrome were possible diagnoses; an exploratory laparotomy was performed, foul-smelling bloody fluid was observed in the peritoneal cavity, stomach exhibited at least 95% necrosis from gastroesophageal junctionto prepyloric region, greater omentum was completely necrotic, posterior abdominal wall was fused to the body of the pancreas. When a partial opening in epiploic transcavity was made, extensive pancreatic necrosis was revealed. Surgical cleaning was performed without gastric resection due to stomach and pancreas involvement.Postoperative care was administered in the ICU with antibiotics, hydration, parenteral nutrition and continuous insulin delivered via an infusion pump. The patient’s conditionworsened in parallel with increases in the inflammatory parameters. An evaluation by hepatobiliary surgery and an abdominal and pelvic contrast using enhanced CT showed a slight increase in volume and the absence of enhancement of the body and tail of the pancreas (Figures 2 A and B) associated with a collection that extended towardthe posterior gastric wall (Figure 2 C), which was found to be thickened and unenhanced (Figures 2 A and C). No involvement of the celiac axis (Figure 2 D) or its main branches was detected. A surgical re-exploration was scheduled for the 8^th^ postoperative day. An abdominal angio-CT was performed and related vascular involvement was ruled out (Figure 3). An exploratory laparotomy revealed abundant, foul-smelling necrotizing free fluid; a culture was taken, surgical cleaning of the cavity was performed. A longitudinal partial gastrectomy of the necrotic body was the decision , and the patient was left with a contained laparotomy (Figure 4). Again, management in the ICU was required with mechanical ventilation for 27 days, after which the patient was transferred to the high-dependency unit, where he remained for 43 days before transfer to a ward. During this period, he was submitted to seven surgical cleanings and required a splenectomy and partial necrosectomy of the tail of the pancreasin addition to various antibiotic therapy regimens. The final surgical cleaning occurred 47 days after the initial one, and at this time, the Bogota bag was removed, and the abdominal wall was closed. Additionally, a high-debit pancreato-digestive fistula was diagnosed and managed with drainage, and acute lithiasic cholecystitis (biliary sludge) was diagnosed and managed via the performance of a percutaneous cholecystostomy.

After 70 days, the patient was transferred to wardwhere he stayed for 36 more days. He progressed to a better general condition, and parenteral nutrition continued to be required due to the presence of the fistula, the debit of which progressively decreased. A Witzel feeding jejunostomy was performed without incident. After 21 days of jejunostomy feeding, a methylene blue test for oral feeding was negative. Therefore, diet by mouth was initiated, with good tolerance and the patient was discharged.

Presently, 17 months after elective discharge, a laparoscopic cholecystectomy plus intraoperative cholangiography (which produced no images suggestive of choledocolithiasis) has been performed, favorable postoperative evolution has been observed. The patient was discharged in good condition with an oral regimen and insulin support.

## DISCUSSION

Understanding our patient’s context first requires an understanding of the pancreatitis classification, which defines three degrees of severity: mild acute, moderately severe acute and severe acute. Constant classification is required due to the dynamic nature of the disease, multidisciplinary management is thus important^7^. The terminology to this classification includes temporary organ failure, persistent organ failure, and local or systemic complications. Organ failure is deemed temporary during the first 48 h and persistent from 48 h on ward^3^ ^,^ ^4^. Local complications include fluid collections and acute necrotic collections, whereas systemic complications can be related to exacerbation of the underlying comorbidities.

In our service, the values used to classify patients as severe are PCR>150 and APACHE >8^5^. This strategy was adopted as a modification of the UK clinical guide that makes more aggressive management possible for patients who present and meet one of these two criteria from admittance up to 48 h^2^. During the past few years, we have introduced the systematic use of the Marshall score upon admittance; however, unlike the other two criteria it has not exhibited any association with mortality, but is associated with admittance to the critical patient unit^6^.

Enteric perforations are a rare complication of acute pancreatitis and involve a severe underlying pathology^7^. This involvement usually occurs in cases of severe necrotizing pancreatitis.

Gastric necrosis related to pancreatitis is a rare complication because the perfusion originates from the branches of the celiac axis^8^. The causes of gastric necrosis can be vascular, toxic, inflammatory, mechanical, infectious, autoimmune or idiopatic^8^. In a case report published in 2012, only two cases were associated with acute pancreatitis^8^ ^,^ ^9^.

Against this background, any vascular complication around the aorta and the celiac axis must be excluded. In this case, the vascular structures were examined via abdominal CT with contrast in the arterial phase (Figure 2 D) and subsequently with abdominal angio-CT, which ruled out pathology of the celiac axis or aorta (Figure 3). Another potential etiology involves the origination of the necrosis from disseminated extravascular coagulation, which would explain why there was no evidence thrombosis detected by the angio-CT.

Another point to emphasize is the rarity of this clinical presentation. In the literature, there is only one case in which a patient with gastric perforation due to pancreatitis initially presented with hematemesis^10^. In another reported case, a perforated gastric ulcer simulated pancreatitis, which emphasizes the importance of imaging to define the etiology^11^.

For this patient, who was in a serious condition that involved multiple organ dysfunction, gastric necrosis in which some vitality of the gastric curvature was preserved, and pancreatic and peripancreatic necrosis, we initially decided to perform a partial gastrectomy and pancreatic necrosectomy and planned several cleanings of the cavity during the evolution. This approach could be consiered “damage control” for severe pancreatitis. It contrasts with the treatments administered in some reports, which include total gastrectomy, esophago-jejunal-anastomosis, left pancreatectomy, cholecystectomy and splenectomy^8^.Subsequent surgeries included a splenectomy for splenic necrosis and pancreatic and peripancreatic necrosectomies. During the evolution, a gastric fistula appeared and subsequently closed spontaneously. To guarantee the closure of this fistula, the feeding jejunostomy was important for nutritional management. Since discharge, endoscopic check-ups have revealed no lesions in the gastric mucosa or stenotic areas.
